# Self-Assembled Carbon Metal–Organic Framework Oxides Derived from Two Calcination Temperatures as Anode Material for Lithium-Ion Batteries

**DOI:** 10.3390/molecules29153566

**Published:** 2024-07-29

**Authors:** Yang Yang, Min Li, Xiaoqin Hu

**Affiliations:** Department of Chemistry, Changzhi University, Changzhi 046000, China; czxylm@126.com (M.L.); xiaoqin_hu2021@126.com (X.H.)

**Keywords:** metal–organic frameworks, calcination, lithium-ion battery

## Abstract

Owing to their structural diversity and mesoporous construction, metal–organic frameworks (MOFs) have been used as templates to prepare mesoporous metal oxides, which show excellent performance as anode materials for lithium-ion batteries (LIBs). Co-ZnO/C and Co-Co_3_O_4_/C nanohybrids were successfully synthesized based on a precursor of Co-doped MOF-5 by accurately controlling the annealing temperature and atmosphere. Experimental data proved that their electrochemical performance was closely associated with the material phase, especially for Co-ZnO/C, indicating that carbon skeleton materials can maintain a good restoration rate of over 99% after undergoing high-current density cycling. Meanwhile, Co-Co_3_O_4_/C nanohybrids showed an exceedingly high reversible capacity of 898 mAh∙g^−1^ at a current density of 0.1 C after 100 cycles. Their improved coulombic efficiency and superior rate capability contribute to a mesoporous structure, which provides pathways allowing for rapid Li^+^ diffusion and regulates volume change during charge and discharge processes.

## 1. Introduction

With the fast growth of new energy vehicles (NEVs, replacing fossil fuel vehicles) around the world, mainly including battery electric vehicles (BEVs), hybrid electric vehicle (HEVs), and fuel cell electric vehicles (FCEVs), the durability of batteries needs to be improved, which is a significant and urgent challenge in the NEV marketplace [1]. Energy storage and conversion based on traditional electrode materials were realized by the oxidation–reduction reaction of transition metal ions and the reversible embedding–stripping process of lithium ions in crystal lattices. In general, graphitic carbon only allows Li^+^ de-intercalation/intercalation from/into their lattices during the charge/discharge process, which leads to a low theoretical specific capacity of 372 mAh∙g^−1^ [2]. Some high-capacity anodes, such as silicon (4200 mAh∙g^−1^) [3], germanium (1623 mAh∙g^−1^) [4], tin (993 mAh∙g^−1^) [5], and so forth, have been investigated; however, these materials present poor electrochemical reversibility, accompanied by severe attenuation in electrochemical cycles. Therefore, electrode materials with high energy density, high safety, and a long cycle life are required for high-performance LIBs.

Preparing electrode materials with complex porous structures is an effective way to improve their capacity and cycling life. Compared with traditional porous materials, metal–organic frameworks (MOFs) are characterized by adjustable pore size, an abundant pore structure, high specific surface area, and a large number of surface functional groups [6]. The metal oxides derived from MOFs not only provide a large number of pores to ensure the diffusion of Li^+^ and, therefore, make full use of active substances but also offer numbers of sites for redox reactions at the ultra-high surface area, as well as the carbon skeleton. Moreover, the internal hollow structure can provide enough space to buffer the mechanical stress caused by the volume change in the cycling processes, preventing the pulverization of electrode materials and dissolution in the electrolyte. Under a simple calcination approach, Fe-MOFs were formed as an ideal carbon cladding iron oxide, which delivered a capacity of 911 mAh∙g^−1^ [7]. The capacity of hierarchically porous carbon-coated ZnO quantum dots, which were obtained by heat treatment, in the IRMOF-1 was reported to be as high as 1200 mAh∙g^−1^ [8]. Various data have reported that MOF-derived transition metal oxides and sulfides such as ZnO, ZnO/ZnFe_2_O_4_, TiO_2_, Co_3_O_4_, CoS, and NiS exhibited good performances when applied in LIBs [9,10,11,12,13,14]. Thus, designing and synthesizing new composite nanostructures in combination with MOFs can alleviate the conductivity and circularity of transition metal oxides due to the fact that the irreversible deformation of the materials is crucial for improving the electrochemical stability of LIBs.

In this study, cobalt was selected to be embedded in the polyhedral porous carbon framework to improve the rate performance and cycling stability of the active material [15], with a convenient and MOF-derived self-template strategy for the synthesis of Co-ZnO/C framework composite material. In addition, with further increase in calcination temperature to 1000 °C, ZnO could be removed in the composites; then, after oxidizing the Co/C composite in air, a nanocomposite of Co-Co_3_O_4_/C could be obtained. Both Co-ZnO/C and Co-Co_3_O_4_/C exhibit remarkable electrochemical stability and high capacities when tested as anode materials for LIBs. Moreover, this synthetic strategy is quite simple, cost-effective, and environmentally friendly, which is highly promising for mass production.

## 2. Results and Discussion

### 2.1. Structure and Morphology Characterization

**Scheme 1:**
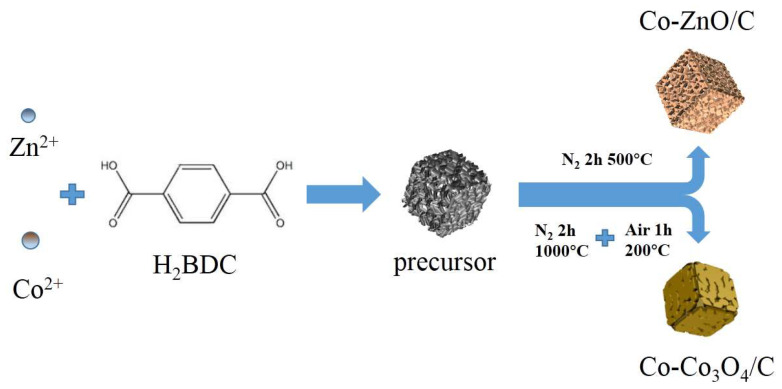
Schematic illustration of the synthesis of Co-ZnO/C and Co-Co_3_O_4_ composites.

The synthetic processes of Co-ZnO/C and Co-Co_3_O_4_/C nanocomposites are described in Scheme 1. First, the Co-doped MOF-5 precursor was synthesized with cobaltous or zinc nitrate hexahydrate and terephthalic acid, which were subsequently mixed with PVP, DMF, and ethanol. Then, the MOF-derived products, Co-ZnO/C and Co-Co_3_O_4_/C, were obtained via a simple annealing treatment of Co-doped MOF-5 precursor under N_2_ and/or air atmospheres. Then, the products obtained after heating at 500 and 1000 °C were Co-ZnO/C and Co-Co_3_O_4_/C with a similar surface morphology. According to previous studies in the literature, ZnO derived from MOF-5 would be reduced if the temperature was over 800 °C, and zinc metal would evaporate at 1000 °C with flowing N_2_ gas, considering the boiling point of zinc metal (908 °C) [16]. Therefore, 1000 °C was selected as the calcination temperature to prepare the zinc metal-free porous carbon structures in which cobalt was partially oxidized in situ to form Co-Co_3_O_4_/C nanoparticles.

**Figure 1:**
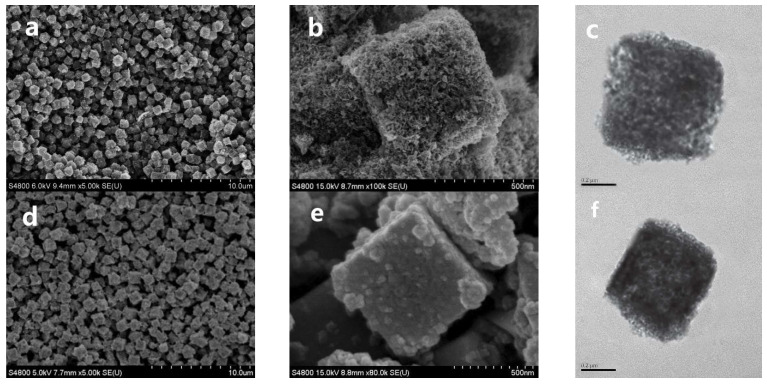
(**a**,**b**) SEM images of Co-ZnO/C and (**c**)TEM image of Co-ZnO/C; (**d**,**e**) SEM images of Co-Co_3_O_4_/C and (**f**) TEM image of Co-Co_3_O_4_/C.

The morphology of the preparative samples relied on field-emission scanning electron microscopy (SEM) and transmission electron microscopy (TEM) measurements which are shown in Figure 1. As shown in Figure 1a–e, Co-ZnO/C and Co-Co_3_O_4_/C reveal carbon nano-cube frames of 500–800 nm inside length. Although by annealing at different temperatures, the frameworks of the carbon nanoparticles were still retained. The size of a part of the nanoparticles became a little bigger owing to the growth of cobalt and cobalt oxide, even during the oxidation process of cobalt nanoparticles. A high magnification image showed that Co-ZnO/C displayed villiform morphology with a wrinkled and transparent structure, indicating a large specific surface area (Figure 1b). Furthermore, to view the internal structure, it can be clearly observed that Co-ZnO/C nanoparticles in the TEM image (Figure 1c) grow uniformly in the carbon frameworks to form sheet nanoporous and hollowed cells which have larger surface areas than the traditional negative electrode materials, could accommodate volume expansion, and also provide more pathway for Li^+^ ions. After heating at 1000 °C, the nanoparticles on the carbon skeleton were slightly agglomerated, causing breakage of the framework, as shown in Figure 1e. However, considering that the zinc-containing material had been evaporated, it distinctly maintained porous surfaces and a hollowed skeleton, which is displayed in the high-resolution TEM image (Figure 1f). The prepared nano-sheet framework active material plays an important role in the performance of LIBs.

**Figure 2:**
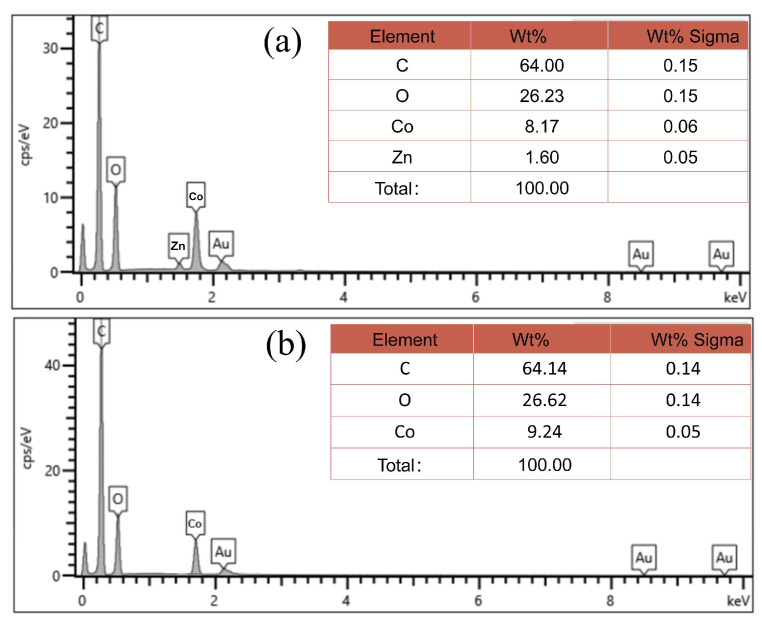
EDS mapping spectra and elemental distributions of (**a**) Co-ZnO/C and (**b**) Co-Co_3_O_4_/C.

Further, the distributions of zinc and cobalt are evident from the EDS mapping spectra and elemental distributions, shown in Figure 2. Both the zinc and cobalt elements are present in Figure 2a with percentage contents of 1.6% and 8.17%, respectively. Whereas Figure 2b shows that no zinc is detected in the material, which confirms the removal of zinc from Co-Co_3_O_4_/C. The percentage content of elemental cobalt is 9.24%.

**Figure 3:**
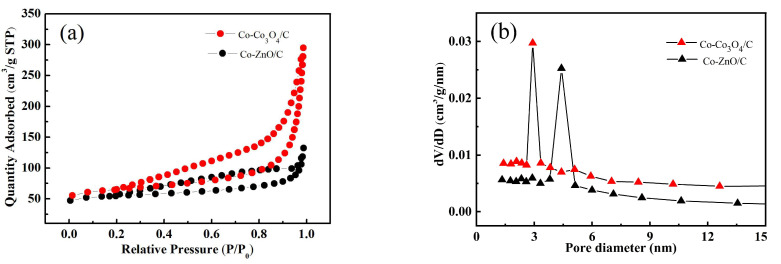
(**a**) Nitrogen sorption–desorption isotherms and (**b**) pore-size distribution of Co-ZnO/C and Co-Co_3_O_4_/C.

By means of Brunauer–Emmett–Teller (BET) to calculate the specific surface areas, both Co-ZnO/C and Co-Co_3_O_4_/C perform the type-IV N_2_ adsorption–desorption isotherm indicating a mesoporous characteristic (Figure 3a) with the specific surface area of Co-ZnO/C 169.9 m^2^/g and Co-Co_3_O_4_/C 192.2 m^2^/g, respectively. In contrast to the specific surface area, the pore size distribution displayed in Figure 3b shows that Co-ZnO/C is distributed at 5 nm and Co-Co_3_O_4_/C at 3 nm. The reduction of pore size in Co-Co_3_O_4_/C nanoparticles was related to agglomeration at a high temperature. Still, it seems to have little effect on the integrated charging–discharging process and the effective volume provided for the diffusion of Li^+^, although the pore diameter increases from 3 nm to 5 nm [17]. The internal hollow structure can provide enough space to buffer the mechanical stress caused by the volume change in the cycling processes, preventing the pulverization of electrode materials and dissolution in the electrolyte. In order to analyze legibly the phase composition of nanostructures, the components are expressed in the XRD patterns.

**Figure 4:**
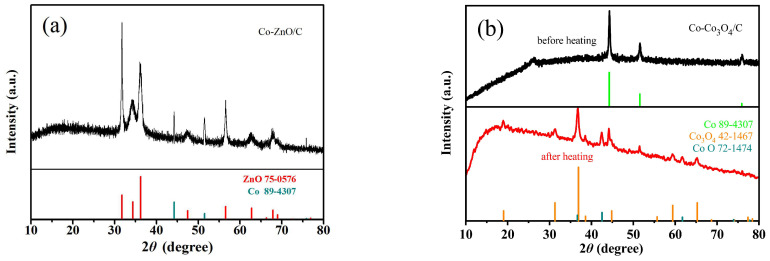
XRD images of (**a**) Co-ZnO/C and (**b**) Co-Co_3_O_4_/C before and after heating conditions.

The XRD pattern of synthesized Co-ZnO/C is illustrated in Figure 4a, which matches well with the theoretical standard peaks of Co (JCPDS, No. 89-4307) and ZnO (JCPDS, No. 75-0576), indicating good phase purity and crystalline quality. The six obvious diffraction peaks of ZnO at 2*θ* = 31.77, 34.37, 36.21, 47.48, 56.53, and 62.77 are consistent with ZnO (100), (002), (101), (002), (110), and (103), respectively. Simultaneously, the three obvious diffraction peaks of Co at 2*θ* = 42.47, 51.52, and 75.85 are in good agreement with Co (111), (200), and (220). The above results indicate that the nanocomposite is a polycrystalline structure with hexagonal-Zn and cubic-Co [18]. However, the XRD pattern of Co-Co_3_O_4_/C, which is not sequentially oxidized in the air at 200 °C for 1 h (top of Figure 4b), displays diffraction peaks at *2θ* = 42.47, 51.57, and 78.54 corresponding Co (111), (200), and (222) that were consistent with cube-shaped Co cells. Moreover, no diffraction peaks of ZnO (peaks at *2θ* = 31.77, 34.38, 36.21, 56.53, 62.77, and 67.86) were found in Figure 4b, suggesting that zinc oxide had been completely decomposed and vaporized, the same as the previous literature [19]. After thermal annealing at 200 °C in air, Co-Co_3_O_4_/C crystallite is oxidized to a mixed cobalt compound shown at the bottom of Figure 4b with four peaks clearly appearing at 2*θ* = 19.1, 31.3, near 36.8, and 42.5 which verified that the cobalt oxides including Co_3_O_4_ (JCPDS No. 42-1467) and CoO (JCPDS No. 72-1474) were obtained after thermal treatment in air owing to different times and temperatures [20,21].

**Figure 5:**
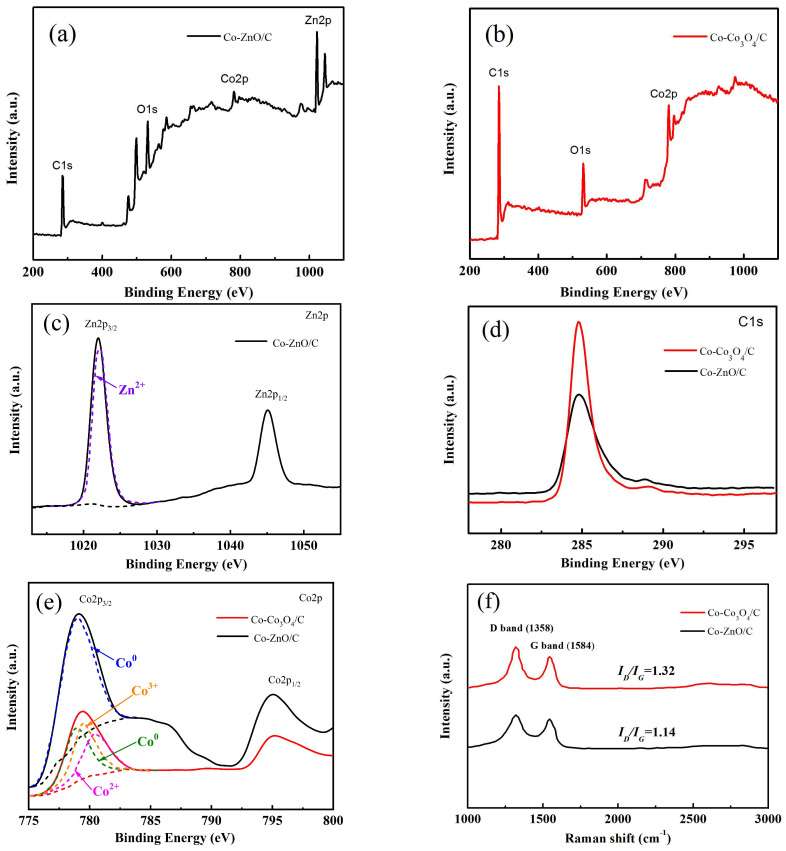
(**a**,**b**) Full spectrum XPS spectra of Co-ZnO/C and Co-Co_3_O_4_/C, respectively, (**c**) Zn 2p, (**d**) C1and (**e**) Co2p XPS spectra of Co-ZnO/C and Co-Co_3_O_4_/C, (**f**) Raman spectrum of Co-ZnO/C and Co-Co_3_O_4_.

In order to investigate the element state of Co-ZnO/C and Co-Co_3_O_4_/C, XPS spectra were analyzed and the full spectral data are shown in Figure 5a,b, respectively. All the binding energies in the XPS spectra were corrected by the C1s peak set at 284.6 eV. In Figure 5a, the characteristic elemental peaks of cobalt, zinc, oxygen, and carbon are evident, while the zinc element is inexistent in Figure 5b; the results agree well with those obtained from the XRD patterns. The high-resolution Zn 2p spectrum (Figure 5c) of Co-ZnO/C nanocomposite denotes that there are only two peaks at 1021.9 eV and 1045.1 eV representing Zn 2p^3/2^ and Zn 2p^1/2^ symmetric peaks of divalent zinc ion [22,23]. A deconvolution analysis of Zn 2p^3/2^ reveals that only one peak at 1022.4 eV for 2p^3/2^ was attributed to its oxide [24]. The main peak of C 1s at 284.8 eV corresponded to the carbon framework sp^2^, as shown in Figure 5d, indicating C atoms in both nanoparticles were conjugated [25]. Furthermore, the high-resolution Co 2p spectrum of Co-ZnO/C (Figure 5e) could be divided into two peaks at 779.9 eV (Co2p^3/2^) and 795.6 eV (Co2p^1/2^) with 15.71 eV difference value, which could be assigned to the splitting energy of metallic Co spin-orbit [26,27]. Dissimilarly, the broad main peak of Co 2p in Co-Co_3_O_4_/C presenting around 780 eV is attributed to Co^2+^ and Co^3+^ from CoO and Co_3_O_4_ phases [28]. The small peak at 795.5 eV demonstrates the existence of metallic Co. These results confirm the presence of Co-atom species in MOF-5 [29]. In Figure 5e, the presence of deconvoluted peaks for Co 2p^3/2^ at 778.5 (Co^0^) [30], 779.5 (Co^3+^), and 780.6 eV (Co^2+^) [31] indicates some partially oxidized cobalt species in Co-Co_3_O_4_/C. In contrast, a peak located at 778.5 eV (Co^0^) represents successful restoration of cobalt by forming Co-ZnO/C. In addition, Figure 5f shows the Raman spectra of Co-ZnO/C and Co-Co_3_O_4_/C, which are carbon materials derived at different calcination temperatures. It could be observed that there were two peaks at 1345 (D band) and 1584 cm^−1^ (G band). Concretely, the D band represented the sp^3^ hybridization of the tetrahedral structure and disordered carbon, and the G band meant the sp^2^ hybridization of the planar structure and ordered carbon. According to the ratio of the intensity of the D band to the G band (I_D_/I_G_), the degree of material defects and heteroatoms were evaluated in the I_D_/I_G_ of Co-ZnO/C and Co-Co_3_O_4_/C as 1.14 and 1.32, respectively, indicating abundant defect sites of carbon networks in Co-Co_3_O_4_/C compared to Co-ZnO/C which provided space for lithium-ion storage and the possibility of improving its electrochemical performance [32]. 

### 2.2. Electrochemical Analysis

**Figure 6:**
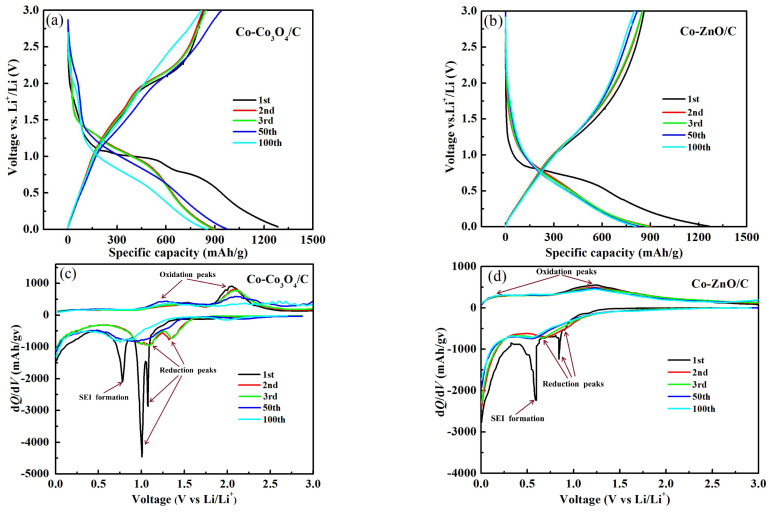
(**a**,**b**) Discharge–charge voltage profiles of Co-Co_3_O_4_/C and Co-ZnO/C in 1st, 2nd, 3rd, 100th cycles. (**c**,**d**) Derivation of the charge–discharge capacity of Co-Co_3_O_4_/C and Co-ZnO/C.

The nanocomposites were also tested as anode materials for LIBs. Figure 6a,b shows the representative galvanostatic discharge/charge curves of Co-ZnO/C and Co-Co_3_O_4_/C for the 1st, 2nd, 3rd, 50th, and 100th cycles with a voltage range of 0.005–3 V (vs. Li/Li^+^) at a current density of 0.1 C. The initial discharge and charge capacities were recorded to be 1285 and 830 mAh∙g^−1^ for Co-ZnO/C and 1270 and 862 mAh∙g^−1^ for Co-Co_3_O_4_/C, respectively. The corresponding initial coulombic efficiencies of 65% and 68% were obtained due to the irreversible process, including the electrolyte contacting with a solid electrode to form an irreversible solid electrolyte interface (SEI) layer and part electrolyte decomposition [33]. From the second cycle onwards, the coulombic efficiency of Co-ZnO/C and Co-Co_3_O_4_/C remained over 90%. Further, to demonstrate distinctly the mechanism of the two nanoparticles, Co-ZnO/C and Co-Co_3_O_4_/C, derivation of the charge–discharge curves are displayed in Figure 6c,d, respectively. Combining Figure 6a,c, three pronounced peaks of Co-Co_3_O_4_/C could be observed at 1.1 V, 1.0 V, and 0.75 V during the initial discharge, which was caused by the formation of the SEI layer and the reduction reaction of Li^+^ embedded CoO and Co_3_O_4_ crystals to form Li_2_O and Co as follows [34]:

Co_3_O_4_ + 8Li^+^ + 8*e^−^* → 3Co + 4Li_2_O (at 1.0 V) (1)

CoO + 2Li^+^ + 2*e^−^* → Co + Li_2_O (at 1.1 V) (2)

The obvious peak of 2.2 V mainly resulted from Li_2_O de-lithiation in the first cycle:

Co +Li_2_O → CoO + 2Li^+^ + 2*e^−^* (at 2.2 V) (3)

In the second cycle, two platforms still occurred with a slight deviation, which was led by the polarization of the electrode [35]. It was worth noting that a weak peak appeared at 1.24 V in every de-lithiation cycle due to CoO oxidation:

3CoO +Li_2_O → Co_3_O_4_ + 2Li^+^ + 2*e^−^* (at 1.24 V) (4)

In the 50th cycle of the lithiation process, there was a broad peak around 1.0 V compared to the 100th cycle which only had a peak at 0.75 V; the master redox mechanism could be considered as mechanism (1) which corresponded well to the de-lithiation process with a gradual gentle peak at 2.2 V which then disappeared in the 100th cycle. Meanwhile, the peak at 1.24 V in the 50th cycle was more pronounced than in the 100th. In contrast to Co-Co_3_O_4_/C, the mechanism of Co-ZnO/C was relatively simple as previous related investigations, so only three peaks at 0.93 V, 0.62 V, and 1.35 V are displayed in Figure 6d during the first discharge–charge cycle, signifying the embedding process of Li^+^ ions and the formation of the SEI and ZnO production, respectively. Lithiation/de-lithiation in the first cycle [36] is as follows:

ZnO + 2Li^+^ + 2*e^−^* ↔ Zn + Li_2_O (at 0.62 V) (5)

However, in the second charge cycle, there were two reactions shifted to 0.9 V and 0.2 V due to the structural reorganization, new phase formation, and polarization in the electrode materials which compared with the standard electrode potential of Co (0.28 V) and Zn (0.76 V), respectively, during the discharge process as follows:

2Co + 2Li_2_O → 2CoO +4Li+ 4*e^−^* (at 0.28 V) (6)

Zn + Li_2_O → ZnO + 2Li^+^ + 2*e^−^* (at 0.76 V) (7)

The differential curves essentially overlap after three cycles, making the peaks at 0.2 V and 0.9 V less visible. Simultaneously, the peaks become flat as a result of the crystalline Co and Zn, progressively changing into smaller particles with an amorphous structure after several reaction cycles. The preceding discharge–charge profiles of the second and third cycle almost overlap with each other, indicating a stable electrochemical performance of the negative electrode. Meanwhile, a relatively high-capacity response occurred near 0 V, which is ascribed to the presence of high amorphous carbon Li^+^ embedded in the product [37].

**Figure 7:**
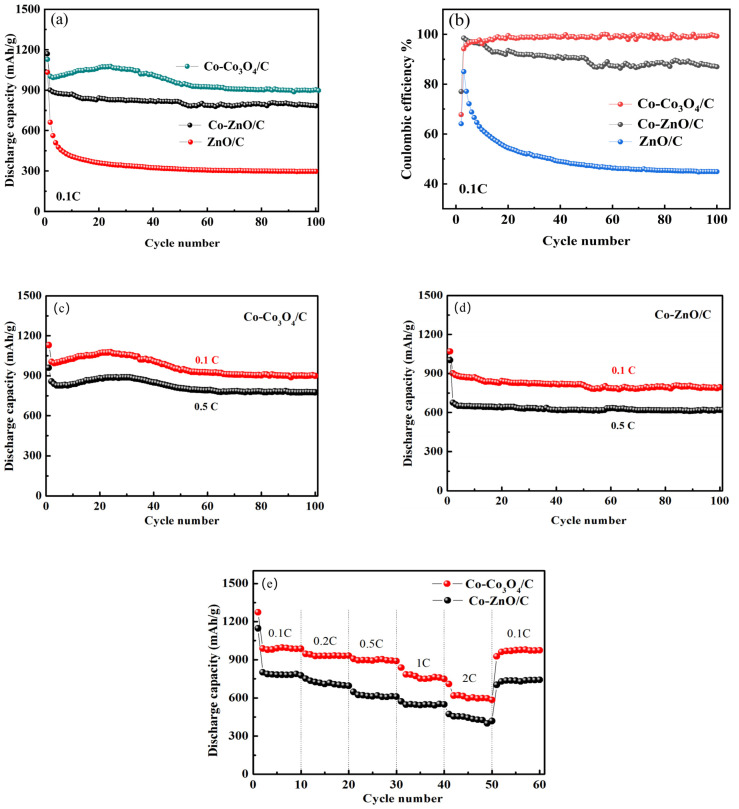
(**a**,**b**) Cycling performance and coulombic efficiency of ZnO/C, Co-ZnO/C and Co-Co_3_O_4_/C at the current density of 0.1 C. (**c**,**d**) Cycling performance of Co-Co_3_O_4_/C and Co-ZnO/C at different current densities of 0.1 C and 0.5 C, respectively. (**e**) Rate performance of Co-Co_3_O_4_/C and Co-ZnO/C electrodes at different rate current densities.

Compared with the cycling performance of ZnO/C, Co-ZnO/C exhibits a higher initial discharge capacity of 1170 mAh∙g^-1^ in Figure 7a, and the capacity falls to 784 mAh∙g^-1^ after 100 cycles. The coulombic efficiency is 87%, as shown in Figure 7b. The proportion of discharge capacity to charge capacity is called coulombic efficiency. It is also named charge–discharge efficiency. In each cycle of charge and discharge, a battery with a higher coulombic efficiency will lose less capacity and have a longer battery life. In Figure 7a, Co-Co_3_O_4_/C has the highest capacity of the three with 1128 mAh∙g^-1^ and fades to 898 mAh∙g^-1^ after 100 cycles. However, the reversible capacity of a certain section gradually increases during the cycle, and the coulombic efficiency gradually increases from the second cycle and remains close to 100% in Figure 7b. The gradual activation process of the metal oxide electrodes may be responsible for the notable improvement in the capacity of Co-ZnO/C and Co-Co_3_O_4_/C over ZnO/C. This phenomenon was reported as a common behavior of metal oxides when applied as anode materials for LIBs [10,38]. However, the capacity of ZnO/C does decay with the number of cycles. After 100 cycles the capacity is only 297 mAh∙g^-1^, far less than the former ones, with a coulombic efficiency of only 45%, as displayed in Figure 7a,b. The dominant electrochemical performance of Co-Co_3_O_4_/C is attributed to two causes. First, the nanosheet structure of Co-Co_3_O_4_/C mesopores expands the contact area between the active materials and the electrolyte to allow more Li^+^ to pass through the interface, shortening the diffusion distance of Li^+^ and electrons. Simultaneously, the abundance of mesopores regulates the volume effect generated during the charging and discharging process and relieves the stress generated internally, resulting in high capacity and multiplication performance. Second, both Co and C increase the electrical conductivity and accelerate the diffusion of Li^+^, thereby improving the multiplicity performance and cycling stability.

Uniformly, the Co-Co_3_O_4_/C in Figure 7c shows an identical discharge trend in current density of 0.5 C for this reason, with electrode activation at 5–30 cycles (827–888 mAh∙g^-1^) and finally leaves behind 776 mAh∙g^-1^. Figure 7d displays the uniform discharge trend of Co-ZnO/C at different current densities of 0.1 C and 0.5 C. Apparently, the frame structure of composites carried out a relatively stable cycling performance even at a higher current density of 0.5 C. It was still able to deliver a capacity of 620 mAh∙g^-1^ after 100 discharge and charge cycles, remaining at 72% of the initial reversible capacity (852 mAh∙g^-1^). The coulombic efficiency of subsequent cycles gradually reached 100%, so porous materials with a carbon skeleton structure are recommended as electrodes for a large current density. Rate performance is an important criterion for high-power practical applications. The rate performance of Co-Co_3_O_4_/C and Co-ZnO/C were evaluated, and the data are shown in Figure 7e. The Co-Co_3_O_4_/C electrode delivers charge capacities of 995, 931, 897, 763, 603, and 979 mAh∙g^-1^ and discharge specific capacity of Co-ZnO/C with 786, 720, 622, 556, 447, and 741 mAh∙g^-1^ in the same current densities of 0.1, 0.2, 0.5, 1, 2, and 0.1 C, indicating the superior structural stability of Co-Co_3_O_4_/C and Co-ZnO/C electrodes, whose capacity returns to 979 and 741 mAh∙g^-1^, respectively, after cycling at high current densities. Moreover, Co-ZnO/C seems to maintain a better efficiency of the charge under high current conditions. In addition, Table S1 displays the electrochemical characteristics of metal oxide–MOF materials recently reported in the literature [39,40,41,42,43,44,45]. The Co-Co_3_O_4_/C anode exhibits advantages in both discharge specific capacity and cycling performance, especially in high current conditions, indicating the potential of this multifunctional anode in practical applications.

## 3. Materials and Methods

### 3.1. Material Preparation

**Figure 8:**
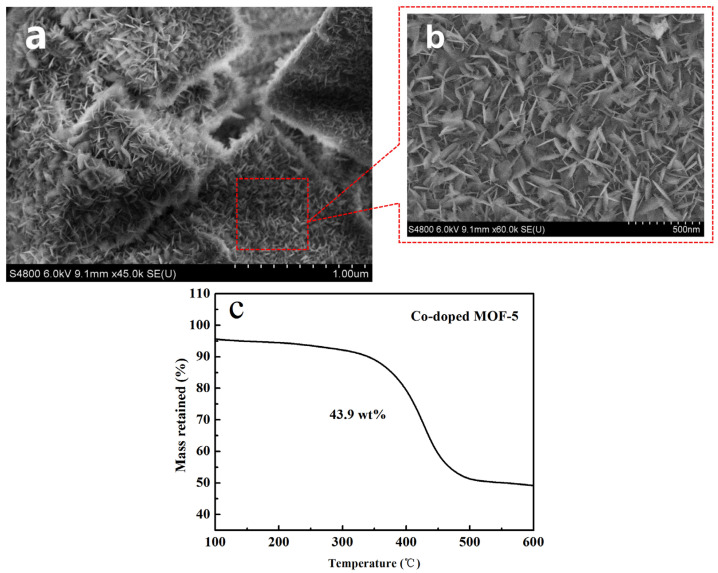
(**a**,**b**) SEM images and (**c**) TG curve of Co-doped MOF-5.

The purity of all chemicals used in the experiments was analytical grade. The morphology of the precursor, nano-sheet framework structure is shown in Figure 8a,b with sizes of 600–800 nm inside length, and its surfaces comprise about 50 nm of nano–sheets. Co-doped MOF-5 is prepared using oil bath reflux as follows: 1.78 g cobaltous nitrate hexahydrate, 1.67g zinc nitrate hexahydrate, 0.37 g terephthalic acid (H_2_BDC), and 7.2 g polyvinyl pyrrolidone (PVP) were dissolved in a three-necked flask containing 480 mL mixed solvent (DMF/ethanol = 5:3) under magnetic stirring for 10 min to dissolve the reactants completely. The mixed solution was then transferred to an oil bath at 100 °C and reacted for 8 h at reflux to obtain a purple–red suspension, placed and stabilized for 24 h. After centrifugation and thoroughly washing several times with ethanol and DMF, respectively, the product was dried at 80 °C under vacuum for 24 h. To confirm the content of carbon (wt%), as indicated in Figure 8c, the precursor Co-doped MOF-5 was heated in air to give a carbon content of around 43.9 wt% according to the main weight loss between 100 and 500 °C, which was attributed to carbon dioxide generation.

Nanostructured Co-doped ZnO (Co_x_Zn_y_O_z_/C) composites were manufactured by calcining of the precursor at 500 °C under a nitrogen atmosphere. Simultaneously, the precursors were also calcined at 1000 °C under the same conditions to obtain Co/C composites, which were further heated at 200 °C in air for 1 h to harvest the final product, Co-Co_3_O_4_/C nanocomposite.

### 3.2. Material Characterization

The composition and crystal structure of the composite materials were characterized by X-ray diffraction (X’pert Pro Super X-ray diffractometer, Philips, The Netherlands) with 2θ range of 5–80°. The surface morphology of all samples was observed with a high-resolution field-emission scanning electron microscope (SEM, S-4800, Hitachi, Japan). Moreover, a transmission electron microscope (TEM, JEM-1400, JEOL Ltd., Akishima City, Japan) was used for microscopic observation of the internal structure. The Brunauer–Emmett–Teller (BET, ASAP 2020, Micro metrics, Norcross, GA, USA) method was carried out to measure the specific surface area and pore size distribution of materials at 77.35 K. Raman spectra of the samples were recorded on an Xplora (Horiba Jobin Yvon, Longjumeau, France) using an Ar ion laser operating at 532 nm. An XPS spectrometer (Quantum-2000, Physical Electronics, Chanhassen, MN, America) was used to analyze the chemical element and electronic states of composites using Al Kα (hv = 1486.6 eV) as the source of excitation.

### 3.3. Electrochemical Measurements

The prepared composites, conductive carbon black (Super P), and aqueous adhesives (polyacrylonitrile, LA), according to a mass ratio of 7:2:1, were mixed with ultrapure water to obtain a uniform slurry, which was evenly coated on copper foils. After drying at 60 °C for 12 h under vacuum, the copper foils were assembled in button cells in a nitrogen atmosphere glove box. The capacity and cycle performance tests of button cells were carried out at room temperature using a battery test system (BT–5, Xinweier, Shenzhen, China) with a voltage range of 0.01–3.0 V (vs. Li/Li^+^).

## 4. Conclusions

Mesoporous Co-ZnO/C and Co-Co_3_O_4_/C composite materials were prepared via a simple calcination method based on the MOF-5 porous structure. Their composition, microstructure, and electrochemical performance were investigated. The results show that both Co-Co_3_O_4_/C and Co-ZnO/C composites have good electrochemical cycling and stability due to the presence of Co, which increases the electrical conductivity of the composites. Compared with the traditional metal oxide electrodes, the MOF-derived ones not only slow the volume expansion, but the flexible conductive carbon frameworks also benefit from fast ion and electron transportation. This research indicates that MOFs can be used as promising precursors to fabricate metal oxides/carbon composite electrode materials with simple fabrication and superior performance, which is anticipated to achieve large-scale industrial production.

## Data Availability

Data are contained within the article and Supplementary Materials.

## Supplementary Materials

The following supporting information can be downloaded at: https://www.mdpi.com/article/10.3390/molecules29153566/s1, Table S1. Electrochemical performance comparison of previously reported metal oxide–MOF anode materials.
