# Supplementary material for: Self-Assembled Carbon Metal–Organic Framework Oxides Derived from Two Calcination Temperatures as Anode Material for Lithium-Ion Batteries
Source: Molecules. 2024 Jul 29;29(15):3566. doi: 10.3390/molecules29153566 (PMC11314184; doi:10.3390/molecules29153566)
Supplement: Supplementary file 1 [file molecules-29-03566-s001.zip › molecules-3078693-supplementary.pdf]

Supporting Information

Self-Assembled Carbon Metal–Organic Framework Oxides Derived from Two Calcination Temperatures as Anode Material for Lithium-Ion Batteries

Yang Yang *, Min Li and Xiaoqin Hu

Department of Chemistry, Changzhi University, Changzhi 046000, China; czxylm@126.com (M. Li); xiaoqin_hu2021@126.com (X. Hu)

*Correspondence: cyhxyy18@126.com; Tel.: +86-035-5217-8321

Table S1. Electrochemical performance comparison of previously reported metal oxide–MOF anode materials.

Anode materials	MOFs utilized	Current density (C)	Initial Capacity (mAh·g^{-1})	Cycle number	Stable capacity at last cycle (mAh·g^{-1})	Capacity decay per cycle (%)	Ref.
Co_3O_4/TiO_2	ZIF-67	0.5	730	200	642	0.0602	[39]
TiO_2/ZnO/C@CNTs	ZIF-8	0.1	943.41	100	816.8	0.1342	[40]
$SnO_2@Fe_2O_3$	MIL-100	0.1	1908	100	750	0.6069	[41]
Co_3O_4	Co-MOF	0.1	1177.4	200	903.1	0.1165	[42]
$NiFe_2O_4/Fe_2O_3$	MIL-88	0.1	1488.4	100	936.9	0.3705	[43]
CuO/C	Cu-BTC	0.1	758.7	200	505	0.1672	[44]
$CoO/Co_2Mo_3O_8$	ZIF-67	0.1	1352.3	500	947.4	0.0599	[45]
Co-Co_3O_4/C	MOF-5	0.5	959.3	100	776	0.1911	This
Co-ZnO/C	MOF-5	0.5	1002.7	100	620	0.3917	wor

References

[39] Xu W., Cui X., Xie Z., Dietrich G. and Wang Y. Integrated Co_3O_4/TiO_2 composite hollow polyhedrons prepared via cation-exchange metal–organic framework for superior lithium-ion batteries. *Electrochim. Acta* **2016**, *222*, 1021–1028.

[40] Cheng H., Xu G., Zhu C., Alhalili Z., Du X. and Gao G. Porous MOF derived TiO_2/ZnO/C@ CNTs composites for enhancing lithium storage performance. *Chem. Eng. J.* **2023**, *454*, 140454.

[41] Zhang J., Wan J., Wang J., Ren H., Yu R., Gu L., Liu Y., Feng S. and Wang D. Hollow multi-shelled structure with metal–organic–framework-derived coatings for enhanced lithium storage. *Angew. Chem.* **2019**, *16*, 5320–5325.

[42] Chen Y., Wang Y., Yang H., Hui G., Cai X., Guo X., Xu B., Lü M. and Yuan A. Facile synthesis of porous hollow Co_3O_4 microfibers derived-from metal–organic frameworks as an advanced anode for lithium ion batteries. *Ceram. Int.* **2017**, *13*, 9945–9950.

[43] Huang G., Zhang F., Zhang L., Du X., Wang J. and Wang L. Hierarchical $NiFe_2O_4/Fe_2O_3$ nanotubes derived from metal organic frameworks for superior lithium ion battery anodes. *J. Mater. Chem. A.* **2014**, *21*, 8048–8053.

[44] Sun Y., Zhang P., Wang B., Wu J., Ning S., Xie A. and Shen Y. Hollow porous CuO/C nanorods as a high-performance anode for lithium ion batteries. *J. Alloys Compd.* **2018**, *750*, 77–84.

[45] Zhao X., Xu H., Hui Z., Sun Y., Yu C., Xue J., Zhou R., Wang L., Dai H., Zhao Y., Yang J., Zhou J., Chen Q., Sun G. and Huang W. Electrostatically assembling 2D nanosheets of MXene and MOF-derivatives into 3D hollow frameworks for enhanced lithium storage. *Small* **2019**, *47*, 1904255.
